# Relationship between perioperative oncological evaluation and recurrence using circulating tumor DNA with *KRAS* mutation in patients with colorectal cancer

**DOI:** 10.1002/cam4.4677

**Published:** 2022-03-21

**Authors:** Takaomi Hayashi, Yoichiro Yoshida, Teppei Yamada, Keita Tanaka, Hideki Shimaoka, Ryuji Kajitani, Taro Munechika, Hideki Nagano, Yoshiko Matsumoto, Akira Komono, Ryohei Sakamoto, Naoya Aisu, Gumpei Yoshimatsu, Fumihiro Yoshimura, Suguru Hasegawa

**Affiliations:** ^1^ Department of Gastroenterological Surgery Fukuoka University Faculty of Medicine Fukuoka Japan

**Keywords:** circulating tumor DNA, colorectal cancer, digital PCR, *KRAS*, liquid biopsy

## Abstract

**Background:**

The detection of circulating tumor DNA (ctDNA) in colorectal cancer (CRC) by liquid biopsy may have prognostic information. In this perioperative study, we evaluate if there is a relationship between mutant allele frequency (MAF) of Kirsten rat sarcoma viral oncogene homolog (*KRAS*) and tumor recurrence and how that could be useful in the early detection of recurrence.

**Methods:**

Among 304 cases of colorectal cancer surgery, ctDNA was sampled from the perioperative blood of 84 patients with CRC with *KRAS* mutation (exon 4 p.A146T, exon 4 p.A146V, exon 2 p.G12A, exon 2 p.G12C, exon 2 p.G12D, exon 2 p.G12S, exon 2 p.G12V, exon 2 p.G13D, exon 3 p.Q61H) and analyzed using the digital polymerase chain reaction system. The median observation period was 26 months.

**Results:**

Although the relationship between the perioperative MAF of *KRAS* and recurrence was not proved, tumor diameter, tumor depth, and stage were correlated with the preoperative MAF of *KRAS* (*p* = 0.034, *p* = 0.002, *p* = 0.008). However, tumor diameter, tumor depth, and stage did not correlate with MAF of *KRAS* at postoperative day 30.

**Conclusions:**

In this study, pathological tumor size, tumor depth, and stage were correlated with preoperative MAF of *KRAS*, but it was unreliable to predict recurrence by detection of ctDNA with *KRAS* mutation in the perioperative period of colorectal surgery.

## Graphical abstract

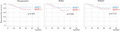

## 1 INTRODUCTION

Colorectal cancer (CRC) is one of the most common cancers. More than 1.8 million new CRC patients and 900,000 deaths expected worldwide in 2020.^1^ The general treatment for nonmetastatic CRC is curative resection and adjuvant chemotherapy according to the stage of cancer. Despite advances in diagnostic imaging, surgery, and chemotherapy, the 5‐year mortality rate remains high at nearly 40%.^2^ Early detection of patients at a higher risk of metastasis after tumor resection is essential in improving clinical outcomes.

Kirsten rat sarcoma viral oncogene homolog *(KRAS)* mutations have been detected in 40–50% of CRC patients, and the mutational status testing has been highlighted recently.^3^, ^4^ *KRAS* is involved in intracellular signaling, mainly in the activation of epidermal growth factor receptor (EGFR) signaling. *KRAS* mutations cause continuous activation of the EGFR intracellular pathway and promote tumor growth and survival, regardless of pharmacological blockade of the EGFR receptor.^4^, ^5^

Conversely, recently, circulating tumor DNA (ctDNA) is considered of high importance as one of the promising biomarkers.^6^ ctDNA is a widely applicable, specific, and sensitive biomarker that can be used for a variety of research and clinical purposes in patients with several types of cancer.^7^ Analyses of biopsy or resected specimens are routinely performed to identify molecular abnormalities that are useful for diagnosis. However, tissue‐based genetic testing does not always fit the current state of cancer due to cancer‐specific characteristics such as clonal evolution over time and heterogeneity within tumors.^8^ Detection and analysis of ctDNA from liquid biopsies, such as blood, saliva, and urine can overcome the above limitations and provide a real‐time and exhaustive characterization of the cancer genome.^9^, ^10^, ^11^, ^12^ Additionally, liquid biopsy is easily repeated and much less invasive than tissue‐based sampling procedures.^13^ However, while blood‐based liquid biopsy has shown great potential for a variety of purposes in several tumor types, it has only been validated in clinical practice for a few selected applications.^14^ To date, liquid biopsy is not considered an alternative to tissue biopsy, which continues to be the standard and indisputable method for diagnosis and biomarker detection of all cancers.

Sequencing technique such as digital polymerase chain reaction (dPCR) is essential for genetic mutation analysis. It offers sensitive sequencing techniques for liquid samples because of its capability to sensitively detect and quantify mutations in small amounts of target DNA.^15^ The detection of circulating tumor cells (CTCs) and ctDNA in serum has been proposed to predict the prognosis of CRC patients as follows. Detection of CTCs postoperatively predict decreased disease‐free survival,^16^ while doing that preoperatively predict early recurrence and decreased disease‐free survival,^17^ and preoperative detection of *KRAS*‐mutated ctDNA is an independent risk factor for recurrence in CRC.^18^ The presence of *KRAS* mutations in ctDNA may reduce survival and affect the efficacy of treatment, especially with cetuximab and panitumumab, but their role in prognosis is still controversial.^19^ A systematic review of 1779 patients concluded that in patients with *KRAS*‐positive colorectal cancer, plasma *KRAS*‐positive status can be a negative prognostic factor in terms of overall survival, progression free survival, and disease‐free survival.^20^ Thirteen of the 17 studies involved patients with stage IV disease, making it a likely biomarker for colorectal cancer patients with metastases. However, the relationship between pre‐ and postoperative changes in ctDNA with *KRAS* mutations and prognosis in patients with curatively resected CRC has not been reported.

This research aims to study the temporal changes of *KRAS* mutation by a liquid biopsy to test the usefulness of measurement of *KRAS* mutation in ctDNA in the perioperative period for predicting recurrence.

## 2 MATERIALS AND METHODS

### 2.1 Study design and patients

This is a prospective study with a median observation period of 26 months. The patients of interest were those who underwent colorectal surgery but have no distant metastasis. They were registered in Fukuoka University Hospital (Fukuoka, Japan) between April 2018 and April 2020. A total of 304 with CRC were sent for primary tumor resection and genetic testing. MEBGEN RASKET‐B kit (MBL, Nagoya, Japan) applies the polymerase chain reaction‐reverse sequence‐specific oligonucleotide method. However, we used it for tissue *KRAS*, neuroblastoma RAS viral oncogene homolog (*NRAS)*, and B‐Raf proto‐oncogene (*BRAF*) tests. Additionally, we excluded cases with *KRAS* wild‐type, *NRAS* mutants, *BRAF* mutants, and a minor *KRAS* mutations such as *KRAS‐* exon 3 p.A59T, ‐ exon 2 p.G12R, and ‐ exon 2 p.G13R. The Institutional Review Board of Fukuoka University Faculty of Medicine approved this research (U19.09.001, 2017‐M‐35). Written informed consent was obtained from all patients.

### 2.2 Blood collection procedures

Peripheral blood samples were collected preoperatively and postoperatively on days 1 and 30. We used BD Vacutainer® PPT plasma preparation tube (Becton, Dickinson and Company) to sample 10 ml blood from each patient. The blood was centrifuged at 1100 *g* for 10 min at 4°C within 2 h after collection. Then, the plasma was transferred to microtubes and stored at −80°C until use.

### 2.3 ctDNA extraction from plasma samples

The plasma samples stored at −80°C were recentrifuged at 16 000 *g* for 10 min at 4°C to remove debris. ctDNA was extracted from 1.0 ml plasma using the Maxwell® RSC cfDNA plasma kit (Promega Corporation) and Maxwell® RSC Instrument (Promega Corporation) in accordance with the manufacturer's protocol, as described previously.^21^

### 2.4 Mutation detection by dPCR

The quantity of ctDNA was calculated using the QuantStudio™ 3D Digital PCR System (Applied Biosystems) as previously reported.^21^ Each polymerase chain reaction (PCR) mixture was prepared with 9.0 μl QuantStudio 3D Digital PCR Master Mix, 0.45 μl TaqMan™ assays, and 8.1 μl ctDNA. We loaded 15.0 μl of the 17.1 μl reaction mixture onto a QuantStudio™ 3D Digital PCR 20 K CHIP using the automatic chip loader. The DNA amplification reaction using the ProFlexTM PCR System (Applied Biosystems) is described in Ref. 21. For dPCR, predesigned dual‐probe TaqMan assays for *KRAS* were purchased from ThermoScientific. We covered nine mutations, exon 4 p.A146T, exon 4 p.A146V, exon 2 p.G12A, exon 2 p.G12C, exon 2 p.G12D, exon 2 p.G12S, exon 2 p.G12V, exon 2 p.G13D, and exon 3 p.Q61H. Results were analyzed using QuantStudio 3D Analysis Suite™ Cloud software. Automatic call assignment for each data cluster was manually adjusted if necessary. Two independent researchers blinded to clinical information performed the dPCR data analysis. The results of the assay were reported as mutant allele frequency (MAF), which is defined as the ratio of mutant DNA molecules to the sum of wild‐type and mutant DNA molecules. A sample was considered positive when the MAF value was greater than 0.15%.^21^, ^22^ Plasmid DNA harboring the *KRAS* mutation (GeneArt, Thermo Fisher Scientific) was used to confirm the sensitivity of dPCR for the *KRAS* mutation. We generated plasmid dilutions down to 0.1% *KRAS* exon 2 p.G12D mutation on a wild‐type plasmid DNA background and used the QuantStudio™ 3D dPCR was in high concordance compared with the allele concentration built by plasmid constructs (*R* ^2^ = 0.99, *p* = 0.003). *KRAS* exon 2 p.G12D mutation in all dilutions down to 0.1% was detected as equivalent values using dPCR.

### 2.5 Statistical analysis

We used IBM SPSS Statistics (IBM Japan, Inc.) to do statistical procedures. However, variables were presented using statistical measures such as numbers or median (interquartile range [IQR]). Additionally, Mann–Whitney *U* test was used to compare between quantitative variables. *p*‐values of <0.05 were considered statistically significant.

## 3 RESULTS

### 3.1 Patient characteristics

A total of 304 patients underwent surgery for CRC and genetic testing of resected specimens at our hospital between April 2018 and April 2020 and 163 patients without *KRAS* mutations were excluded (139 wild‐type, 12 *NRAS* mutations, and 12 *BRAF* mutations). Finally, only 141 patients with *KRAS* mutations were considered. Moreover, 57 patients with distant metastasis or minor *KRAS* mutations or double *KRAS* mutations or no liquid biopsy samples were excluded. Eighty‐four patients who had *KRAS* mutations were included in the analysis (Figure S1).

The characteristics of these 84 patients are shown in Table 1. The median age was 70 years (range, 28–90). The Eastern Cooperative Oncology Group performance score (ECOG PS) was 0 in 57 patients, 1 in 20 patients, and ≥2 in seven patients. The median diameter of the pathological primary cancer was 40 mm (range, 6–105 mm). Pathological tumor depth was less than T2 in 27 patients and T3 or greater in 57 patients. The stage was 0.1 in 23 patients, stage II in 34 patients, and stage III in 27 patients. Recurrence was observed in 14 cases (Table S1).

**TABLE 1:** Demographics and characteristics of patients with *KRAS* mutation

		*N* = 84
Sex	Male/Female	49/35
Age (years)		70 (28–90)
BMI (kg/m^2^)	≤20/20<, ≤24/24<	33/30/21
ECOG PS	0/1/2≤	57/20/7
Tumor location	Right‐sided/Left‐sided	21/63
CEA (ng/ml)	3.85 (0.8–82.6)	
CA19‐9 (U/ml)	7 (1.6–110)	
KRAS mutation		
	A146T/A146V	5/3
	G12A /G12C /G12D /G12S/G12V	5/1/32/7/16
	G13D	12
	Q61H	3
Tumor size (mm)		40 (6–105)
	<70/70≤	68/18
Histological grade		
	Well/Moderately/Poorly/Mucinous	67/10/1/6
p‐Tumor depth		
	Tis	2
	T1	9
	T2	16
	T3	38
	T4 (T4a/T4b)	19 (17/2)
p‐Lymph node metastasis		
	N0	57
	N1 (N1a/N1b/N1c)	21 (8/13/0)
	N2 (N2a/N2b)	6 (5/1)
p‐Stage		
	0	2
	l	21
	ll (lla/llb/llc)	34 (24/9/1)
	lll (llla/lllb/lllc)	27 (4/18/5)
Recurrence		14
	Lymph node/Liver/Lung/Peritoneum/Local	2/4/6/3/2

Abbreviations: BMI, body mass index; ECOG PS, The Eastern Cooperative Oncology Group performance score.

### 3.2 Relationship between the detection of KRAS mutations in the perioperative period and recurrence

The correlation between detection of perioperative *KRAS* mutations and recurrence was simplified using a MAF cutoff value equal 0.15 (Table S2). There was no correlation between the detection of *KRAS* mutations in ctDNA preoperatively, on postoperative day 1 (POD1), and on postoperative day 30 (POD30), and the presence of recurrence.

### 3.3 Relationship between clinical factors and MAF of KRAS mutants in the perioperative period

**FIGURE 1:**
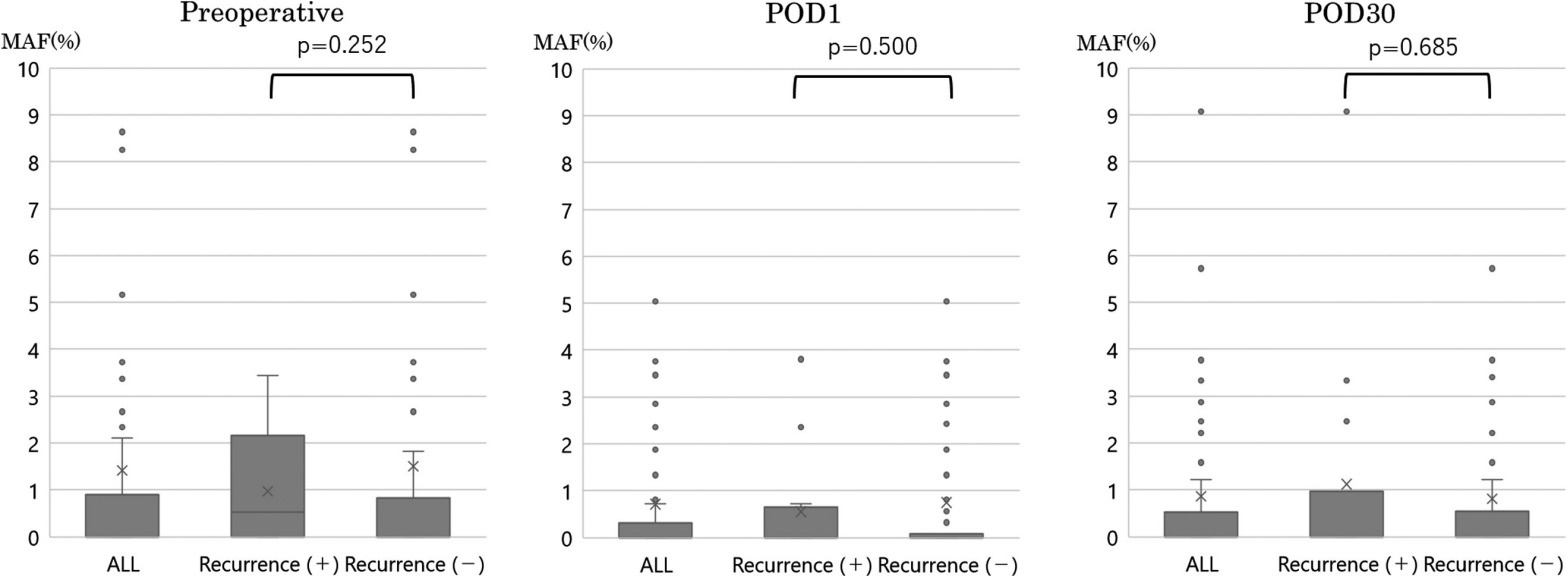
Relationship between the perioperative mutant allele frequency (MAF) of *KRAS* and recurrence. There was no significant difference in MAF at preoperative, postoperative day (POD) 1, and POD 30 between recurrent and non‐recurrent patients (Mann–Whitney U test; *p* =  0.252, *p* = 0.500, and *p* = 0.685, respectively). MAF, mutant allele frequency; POD, postoperative day

**FIGURE 2:**
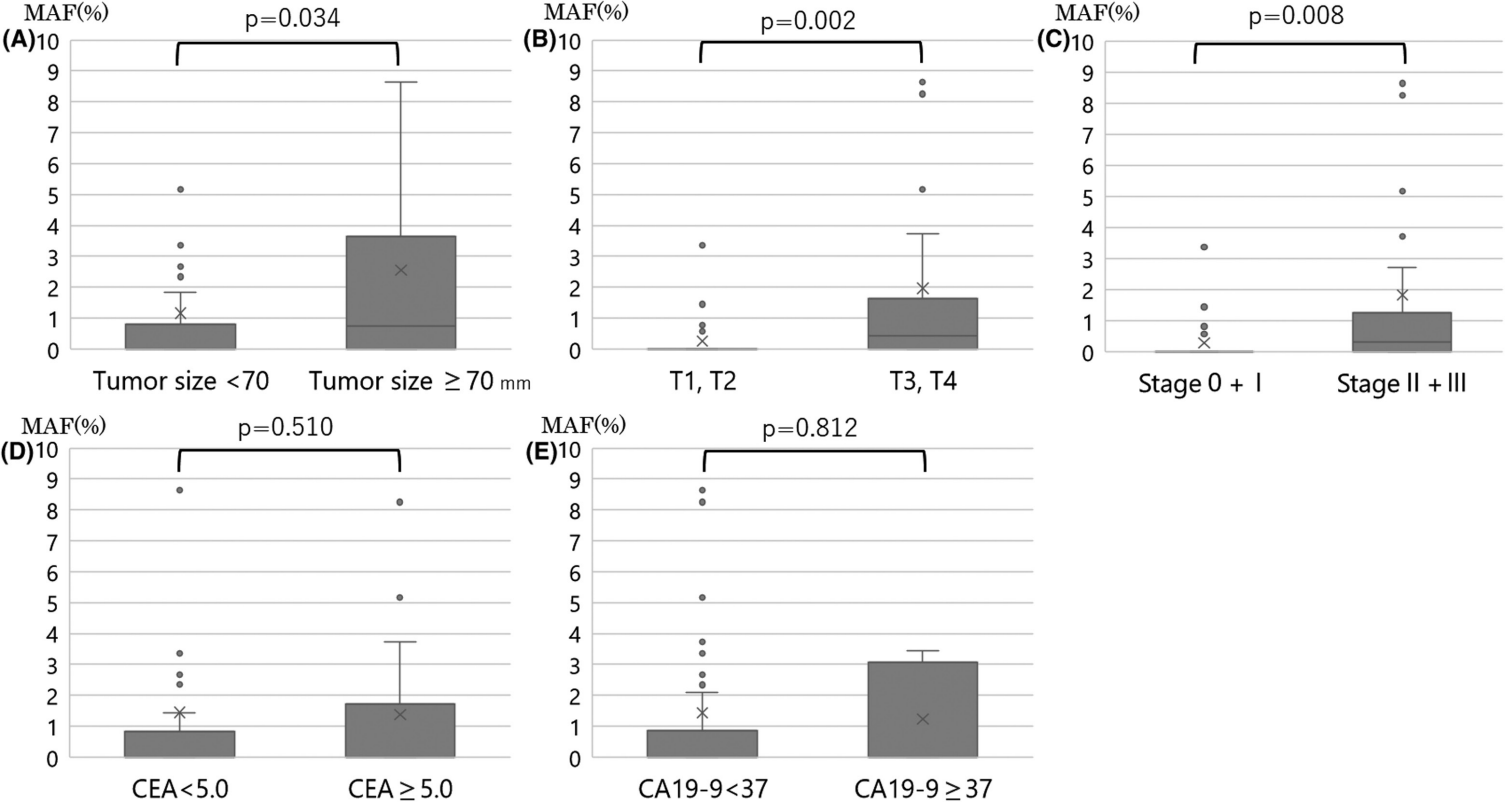
Relationship between preoperative *KRAS* mutant allele frequency and tumor size, tumor depth, stage, and tumor markers. (A) Tumor size, (B) tumor depth (T factor), (C) stage, (D) CEA, and (E) CA19‐9. Pairwise comparisons showed that the larger the tumor size, T factor, stage, the higher the MAF (Mann–Whitney *U* test; *p* = 0.034, *p* = 0.002, *p* = 0.008, respectively). There were no significant differences in CEA level, or CA19‐9 level. (Mann–Whitney *U* test; *p* = 0.510, and *p* = 0.812, respectively). MAF, mutant allele frequency

**FIGURE 3:**
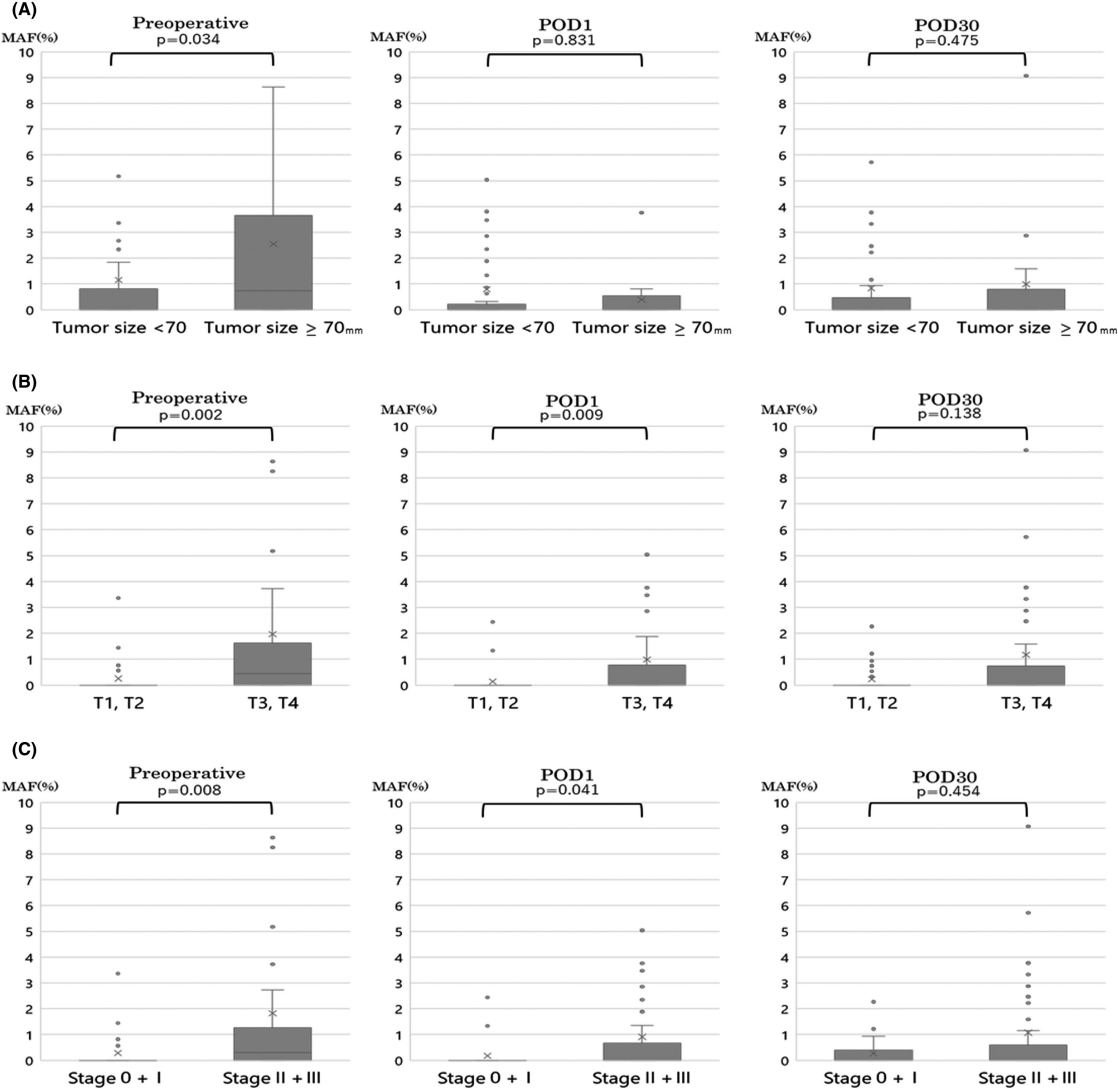
Perioperative *KRAS* mutant allele frequency. (A) Relationship between perioperative *KRAS* mutant allele frequency and tumor size. There was a significant difference in tumor diameter and mutant allele frequency only before surgery. (B) Relationship between perioperative *KRAS* mutant allele frequency and tumor depth (T factor). Significant differences in tumor depth and mutant allele frequency were found in preoperative and POD1. (C) Relationship between perioperative *KRAS* mutant allele frequency and stage. Significant differences in stage and mutant allele frequency were found in preoperative and postoperative day 1. MAF, mutant allele frequency; POD, postoperative day

We examined the correlation between MAF values and recurrence without setting a cutoff value. Even without a cutoff value for the MAF value, the MAF values at preoperative, POD1, and POD30 did not correlate with recurrence (Figure 1). Next, we investigated the relationship between tumor diameter, tumor depth (T factor), stage, and tumor markers (CEA, CA19‐9) and preoperative MAF values (Figure 2). There were significant differences in preoperative MAF values for tumor diameter, tumor depth, and stage. Therefore, we examined whether there was a significant difference in MAF values among preoperative, POD1, and POD30 in those three items. For the tumor diameter, there is a significant difference in preoperative, but no significant difference in POD1 and POD30 (Figure 3A). For tumor depth (Figure 3B) and stage (Figure 3C), there is a significant difference between the preoperative and POD1, but not for POD30.

### 3.4 Relationship between perioperative MAF and recurrence

**FIGURE 4:**
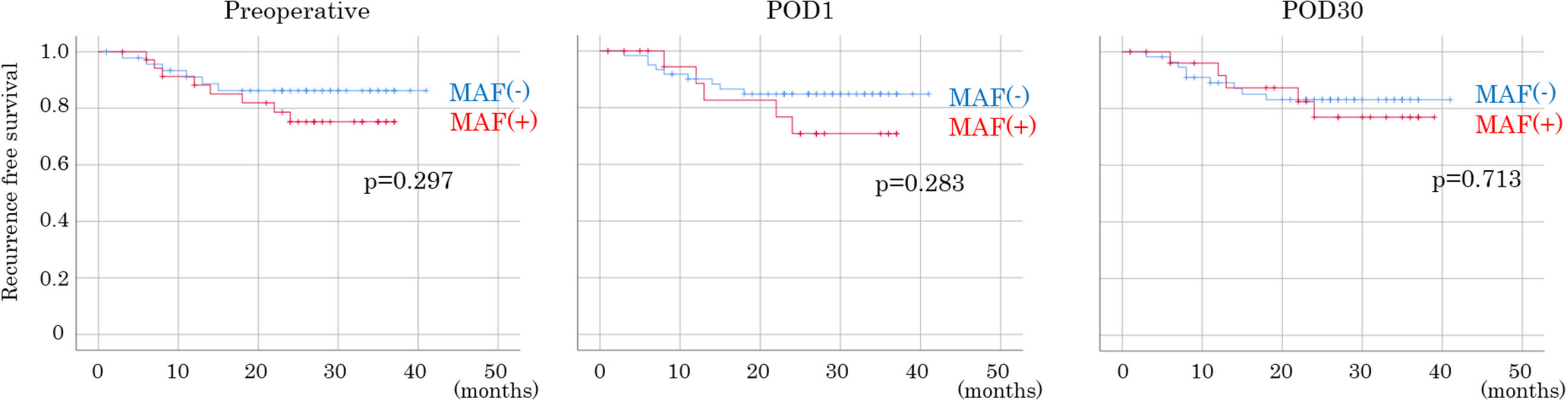
Kaplan–Meier survival curves for recurrence with positive and negative mutant allele frequency. There was no significant difference in mutant allele frequency and recurrence in either preoperative, postoperative day (POD)1, POD30. MAF, mutant allele frequency; POD, postoperative day

The Kaplan–Meier survival curve for recurrence is shown in Figure 4, dividing MAF into positive and negative at the cutoff value. There was no significant difference in MAF and recurrence in either preoperative, POD1, POD30. The MAF (+) and MAF (−) curves are the closest at POD30.

## 4 DISCUSSION

Recent technological advances in ctDNA assays that can detect minimal residual disease after curative surgery have the potential to fundamentally change the assessment of recurrence risk and adjuvant chemotherapy.^23^, ^24^, ^25^, ^26^ However, it is unclear which genetic mutations are the most efficient to target. Previous studies reported that some cancer cells start to appear in the blood coming from the primary tumor at an early stage of cancer, but a very inefficient process must be followed for metastasis to be established.^27^, ^28^, ^29^, ^30^, ^31^ Since the presence of cancer cells in blood is not synonymous with recurrence, the use of specific genetic mutations associated with recurrence will enable effective prediction of recurrence. In the present study, our results showed that *KRAS* mutants in ctDNA did not predict cancer relapse. Therefore, we speculate that cancer recurrence cannot be predicted by *KRAS* mutations in ctDNA alone. There are several reports on the relationship between *KRAS* mutations in ctDNA and recurrence after radical resection of colorectal cancer (Table 2).^18^, ^32^, ^33^, ^34^, ^35^ However, the background of those reports is different. The stage of CRC, the number of *KRAS* mutant gene targets, the method of ctDNA detection, the cutoff value, and the timing of blood collection vary from study to study. So we will specifically compare it to the latest research.

**TABLE 2:** Comparison of the background of studies on the relationship between recurrence and *KRAS* mutants in ctDNA

Author	Year	Pts (Stage≤III, Dukes≤C)	Stage (I/II/III) Dukes (A/B/C)	Pts *KRAS* mt in tissue	Pts *KRAS* mt in ctDNA	Recurrence rate (%)	Targeted *KRAS* mt in ctDNA	Method	Cutoff value	Time of blood collection*
Lecomt	2002	45	I/II/III 8/21/16	22	10	NM	G12A, G12C, G12D, G12R, G12S, G12V, G13A, G13C, G13D, G13R, G13S, G13V	MASA	NM	Pre
Ryan	2003	85	A/B/C 11/53/21	60	16	21.3	Codon 12, codon 13	PCR	NM	Post
Scholer	2017	27	NM	27	NM	55.6	G12A, G12C, G12D, G12S, G12V, G13D, Q61H	dPCR	0.5%	Post
Thomsen	2017	294	I/II/III 40/151/103	107	33	NM	G12D, G12V, G13D,codon 61, codon 117, codon 146	dPCR	0–0.06%	Pre
Nakamura	2021	154	Stage≤III 154	NM	46	14.0	G12A, G12C, G12D, G12R, G12S, G12V, G13D	dPCR	0.02%	Pre
Our study		84	0/I/II/III 2/21/34/27	84	36	16.7	A146T, A146V, G12A, G12C, G12D, G12S, G12V, G13D, Q61H	dPCR	0.15%	Pre, post

* Time of blood collection used for cancer recurrence prediction analysis.

Abbreviations: dPCR, digital polymerase chain reaction; MASA, mutant‐allele‐specific amplification; mt, mutants; NGS, Next Generation Sequencing; NM, not mentioned; PCR, polymerase chain reaction; Pre, preoperative, Post, postoperative.; Pts, patients.

Nakamura et al. reported that *KRAS*‐mutated ctDNA in blood before surgery is related significantly to recurrence after radical resection in patients with CRC.^18^ Our results differ not only from their research results, but also from several other methods. The first is the number of targeted *KRAS* gene mutations. While the previous study covered seven mutations, exon 2 p.G12A, exon 2 p.G12C, exon 2 p.G12D, exon 2 p.G12R, exon 2 p.G12S, exon 2 p.G12V, and exon 2 p.G13D, we covered nine mutations, exon 4 p.A146T, exon 4 p.A146V, exon 2 p.G12A, exon 2 p.G12C, exon 2 p.G12D, exon 2 p.G12S, exon 2 p.G12V, exon 2 p.G13D, and exon 3 p.Q61H. Next is the difference in cutoff values of MAF. We set the cutoff value for MAF at 0.15, while in that study it was 0.02. Therefore, we changed the cutoff value to 0.02; however, the results were the same (data not shown). In addition, the cutoff value was set to every 0.1 from 0.1 to 1.0, but the results were the same. Finally, there is a difference in the *KRAS* status of the primary tumor. We have confirmed the *KRAS* mutations of the primary tumor in all patients, but Nakamura's study did not describe the *KRAS* status of the primary tumor. Furthermore, there was no significant difference between *KRAS* status in ctDNA and lymph node or distant metastasis. Therefore, we believe that further studies are needed to conclude whether the presence of *KRAS* mutant ctDNA before surgery is significantly associated with recurrence after radical resection in patients with CRC.

Using biomarkers to classify cancer patients as high or low risk may help in applying the most appropriate care and treatment within each group. In the case of continuous variable biomarkers, cutoff values need to be established to define the groups. A widespread and simple method is to select a cutoff value that minimizes the P‐value when outcomes are compared between two groups. However, this method suffers from a high false‐positive rate due to multiple testing and a tendency to overestimate the significance of the cutoff value obtained.^36^ Table 2 shows that the methods and cutoff values of these studies are different. To validate our cutoff values, we diluted plasmid down to 0.1% mutation using a wild‐type plasmid DNA background and the QuantStudio™ 3D dPCR System to quantify MAF and plasmid copy numbers.^21^ MAF determined by dPCR was in high concordance compared with the allele concentration built by plasmid constructs (*R* ^2^ = 0.99, *p* = 0.003). A calibration curve was used to confirm that the cutoff value was appropriate for detecting the genetic mutation. There was no significant difference in Kaplan–Meier survival curves for recurrence classified by MAF, either preoperatively, at POD1, or at POD30. As mentioned above, the cutoff value was set to every 0.1 from 0.1 to 1.0, but the results were the same. In the future, it will be necessary to accumulate a large amount of data under the same conditions to set an appropriate cutoff value.

The current study had some limitations. First, the small sample size and usage of the data of one single institution, while multicenter approach is needed. Although there was no significant difference in the results of this study, it seems that liquid biopsy of POD1 may have the potential to predict minimal residual disease. Currently, there is no unified optimal method or cutoff value, and this may be unavoidable. In addition, although MAF was used in this study, it may be better to study using copy numbers. Once those issues are resolved, multicenter clinical trials are desirable. Second, since the observation period in this study was 26 months, there is a possibility that other patients may relapse in the future. Third, only *KRAS* gene mutations were analyzed by dPCR. Cancer is heterogeneous in space and time, and recurrent tumors may not have *KRAS* mutations. In the future, researchers should consider other possible combinations of genetic mutations to examine their ability to predict recurrence.

In conclusion, measuring ctDNA for *KRAS* mutations in the perioperative period is unreliable to predict recurrence early in the current design. As metastasis may represent a very inefficient process, this may lead to difficulty in the early prediction of cancer recurrence.

## CONFLICT OF INTEREST

We have no conflict of interests to declare.

## AUTHOR CONTRIBUTION

**Takaomi Hayashi:** Data collection, data analysis, writing of the manuscript, editing and review of manuscript drafts. **Yoichiro Yoshida:** Conceptualization and design, administrative support, financial support, provision of study materials and/or patients, collection and assembly of data, and data analysis and interpretation. **Teppei Yamada:** Data collection, editing and review of manuscript drafts. **Keita Tanaka:** Provision of research materials and/or patients and collection and assembly of data. **Hideki Shimaoka:** Provision of research materials, collection, and tabulation of data. **Fumihiro Yoshimura:** Provision of study materials and/or patients, and collection and assembly of data. **Ryuji Kajitani:** Provision of research materials, collection, and tabulation of data. **Taro Munechika:** Provision of research materials, collection, and tabulation of data. **Yoshiko Matsumoto:** Provision of research materials, collection, and tabulation of data. **Hideki Nagano:** Provision of research materials, collection, and tabulation of data. **Akira Komono:** Provision of research materials, collection, and tabulation of data. **Ryohei Sakamoto:** Provision of analysis equipment and data collection. **Naoya Aisu:** Provision of research materials, collection, and tabulation of data. **Gumpei Yoshimatsu:** Data analysis and interpretation. **Suguru Hasegawa:** Data analysis and interpretation.

## ETHICAL APPROVAL STATEMENT

The Institutional Review Board of Fukuoka University Faculty of Medicine approved this research (U19.09.001, 2017‐M‐35).

## Supporting information


Figure 1

Table 1

## Data Availability

The datasets analyzed in this study are available from the corresponding author upon request.

## References

[1] Sung H , Ferlay J , Siegel RL , et al. Global cancer statistics 2020: GLOBOCAN estimates of incidence and mortality worldwide for 36 cancers in 185 countries. CA Cancer J Clin. 2021;71:209‐249. 33538338 10.3322/caac.21660

[2] Osterman E , Glimelius B . Recurrence risk after up‐to‐date colon cancer staging, surgery, and pathology: analysis of the entire Swedish population. Diseases of the Colon & Rectum. 2018;61:1016‐1025. 30086050 10.1097/DCR.0000000000001158

[3] Vaughn CP , ZoBell SD , Furtado LV , Baker CL , Samowitz WS . Frequency of KRAS, BRAF, and NRAS mutations in colorectal cancer. Genes Chromosomes Cancer. 2011;50:307‐312. 21305640 10.1002/gcc.20854

[4] Tan C , Du X . KRAS mutation testing in metastatic colorectal cancer. World J Gastroenterol: WJG. 2012;18:5171. 23066310 10.3748/wjg.v18.i37.5171 PMC3468848

[5] Vigil D , Cherfils J , Rossman KL , Der CJ . Ras superfamily GEFs and GAPs: validated and tractable targets for cancer therapy? Nat Rev Cancer. 2010;10:842‐857. 21102635 10.1038/nrc2960 PMC3124093

[6] Han X , Wang J , Sun Y . Circulating tumor DNA as biomarkers for cancer detection. Genomics Proteomics Bioinformatics. 2017;15:59‐72. 28392479 10.1016/j.gpb.2016.12.004 PMC5414889

[7] Bettegowda C , Sausen M , Leary RJ , et al. Detection of circulating tumor DNA in early‐ and late‐stage human malignancies. Sci Transl Med. 2014;6:224ra224. 10.1126/scitranslmed.3007094 PMC4017867 24553385

[8] Gerlinger M , Rowan AJ , Horswell S , et al. Intratumor heterogeneity and branched evolution revealed by multiregion sequencing. N Engl J Med. 2012;366:883‐892. 22397650 10.1056/NEJMoa1113205 PMC4878653

[9] Diaz LA Jr , Bardelli A . Liquid biopsies: genotyping circulating tumor DNA. J Clin Oncol. 2014;32:579‐586. 24449238 10.1200/JCO.2012.45.2011 PMC4820760

[10] Yamada T , Iwai T , Takahashi G , et al. Utility of KRAS mutation detection using circulating cell‐free DNA from patients with colorectal cancer. Cancer Sci. 2016;107:936‐943. 27116474 10.1111/cas.12959 PMC4946708

[11] Ohta R , Yamada T , Sonoda H , et al. Detection of KRAS mutations in circulating tumour DNA from plasma and urine of patients with colorectal cancer. Eur J Surg Oncol. 2021;47:3151‐3156. 34315643 10.1016/j.ejso.2021.07.017

[12] Sclafani F , Chau I , Cunningham D , et al. KRAS and BRAF mutations in circulating tumour DNA from locally advanced rectal cancer. Sci Rep. 2018;8:1445. 29362371 10.1038/s41598-018-19212-5 PMC5780472

[13] Maia MC , Salgia M , Pal SK . Harnessing cell‐free DNA: plasma circulating tumour DNA for liquid biopsy in genitourinary cancers. Nat Rev Urol. 2020;17:271‐291. 32203306 10.1038/s41585-020-0297-9

[14] Russano M , Napolitano A , Ribelli G , et al. Liquid biopsy and tumor heterogeneity in metastatic solid tumors: the potentiality of blood samples. J Exp Clin Cancer Res. 2020;39:95. 32460897 10.1186/s13046-020-01601-2 PMC7254767

[15] Lee KH , Lee TH , Choi MK , Kwon IS , Bae GE , Yeo M‐K . Identification of a clinical cutoff value for multiplex KRASG12/G13 mutation detection in colorectal adenocarcinoma patients using digital droplet PCR, and comparison with sanger sequencing and PNA clamping assay. J Clin Med. 2020;9:2283. 10.3390/jcm9072283 PMC7409004 32708359

[16] Yang C , Shi D , Wang S , Wei C , Zhang C , Xiong B . Prognostic value of pre‐and post‐operative circulating tumor cells detection in colorectal cancer patients treated with curative resection: a prospective cohort study based on ISET device. Cancer Management and Research. 2018;10:4135‐4144. 30323669 10.2147/CMAR.S176575 PMC6177518

[17] Thorsteinsson M , Jess P . The clinical significance of circulating tumor cells in non‐metastatic colorectal cancer–a review. European Journal of Surgical Oncology (EJSO). 2011;37:459‐465. 21324632 10.1016/j.ejso.2011.01.025

[18] Nakamura Y , Yokoyama S , Matsuda K , et al. Preoperative detection of KRAS mutated circulating tumor DNA is an independent risk factor for recurrence in colorectal cancer. Sci Rep. 2021;11:1‐8. 33432066 10.1038/s41598-020-79909-4 PMC7801374

[19] Zhuang R , Li S , Li Q , et al. The prognostic value of KRAS mutation by cell‐free DNA in cancer patients: a systematic review and meta‐analysis. PLoS One. 2017;12:e0182562. 28796802 10.1371/journal.pone.0182562 PMC5552123

[20] Perdyan A , Spychalski P , Kacperczyk J , Rostkowska O , Kobiela J . Circulating tumor DNA in KRAS positive colorectal cancer patients as a prognostic factor–a systematic review and meta‐analysis. Crit Rev Oncol Hematol. 2020;154:103065. 32763752 10.1016/j.critrevonc.2020.103065

[21] Tanaka K , Yoshida Y , Yamada T , et al. Oncological evaluation in the perioperative period using cfDNA with BRAF V600E mutation in patients with colorectal cancer. Sci Rep. 2021;11:1‐8. 34168268 10.1038/s41598-021-92795-8 PMC8225636

[22] García‐Saenz JA , Ayllón P , Laig M , et al. Tumor burden monitoring using cell‐free tumor DNA could be limited by tumor heterogeneity in advanced breast cancer and should be evaluated together with radiographic imaging. BMC Cancer. 2017;17:1‐8. 28330468 10.1186/s12885-017-3185-9 PMC5362993

[23] Tie J , Wang Y , Tomasetti C , et al. Circulating tumor DNA analysis detects minimal residual disease and predicts recurrence in patients with stage II colon cancer. Sci Transl Med. 2016;8:346ra392. 10.1126/scitranslmed.aaf6219 PMC5346159 27384348

[24] Corcoran RB , Chabner BA . Application of cell‐free DNA analysis to cancer treatment. New Engl J Med. 2018;379:1754‐1765. 30380390 10.1056/NEJMra1706174

[25] Chen G , Peng J , Xiao Q , et al. Postoperative circulating tumor DNA as markers of recurrence risk in stages II to III colorectal cancer. J Hematol Oncol. 2021;14:1‐11. 34001194 10.1186/s13045-021-01089-z PMC8130394

[26] Wang Y , Li L , Cohen JD , et al. Prognostic potential of circulating tumor DNA measurement in postoperative surveillance of nonmetastatic colorectal cancer. JAMA Oncol. 2019;5:1118‐1123. 31070668 10.1001/jamaoncol.2019.0512 PMC6512291

[27] Patel H , Le Marer N , Wharton RQ , et al. Clearance of circulating tumor cells after excision of primary colorectal cancer. Ann Surg. 2002;235:226‐231. 11807362 10.1097/00000658-200202000-00010 PMC1422418

[28] Phallen J , Sausen M , Adleff V , et al. Direct detection of early‐stage cancers using circulating tumor DNA. Sci Transl Med. 2017;9:1‐12. 10.1126/scitranslmed.aan2415 PMC6714979 28814544

[29] Weiss L . Metastatic inefficiency. Adv Cancer Res. 1990;54:159‐211. 1688681 10.1016/s0065-230x(08)60811-8

[30] Fidler IJ . Metastasis: quantitative analysis of distribution and fate of tumor emboli labeled with 125I‐5‐iodo‐2′‐deoxyuridine. J Natl Cancer Inst. 1970;45:773‐782. 5513503

[31] Luzzi KJ , MacDonald IC , Schmidt EE , et al. Multistep nature of metastatic inefficiency: dormancy of solitary cells after successful extravasation and limited survival of early micrometastases. Am J Pathol. 1998;153:865‐873. 9736035 10.1016/S0002-9440(10)65628-3 PMC1853000

[32] Lecomte T , Berger A , Zinzindohoué F , et al. Detection of free‐circulating tumor‐associated DNA in plasma of colorectal cancer patients and its association with prognosis. Int J Cancer. 2002;100:542‐548. 12124803 10.1002/ijc.10526

[33] Ryan B , Lefort F , McManus R , et al. A prospective study of circulating mutant KRAS2 in the serum of patients with colorectal neoplasia: strong prognostic indicator in postoperative follow up. Gut. 2003;52:101‐108. 12477769 10.1136/gut.52.1.101 PMC1773535

[34] Schøler LV , Reinert T , Ørntoft M‐BW , et al. Clinical implications of monitoring circulating tumor DNA in patients with colorectal cancer. Clin Cancer Res. 2017;23:5437‐5445. 28600478 10.1158/1078-0432.CCR-17-0510

[35] Thomsen CEB , Appelt AL , Andersen RF , Lindebjerg J , Jensen LH , Jakobsen A . The prognostic value of simultaneous tumor and serum RAS/RAF mutations in localized colon cancer. Cancer Med. 2017;6:928‐936. 28378527 10.1002/cam4.1051 PMC5430097

[36] Woo SY , Kim S . Determination of cutoff values for biomarkers in clinical studies. Precis Future Med. 2020;4:2‐8.

