# Supplementary figures and images for: Relationship between perioperative oncological evaluation and recurrence using circulating tumor DNA with KRAS mutation in patients with colorectal cancer
Source: Cancer Med. 2022 Mar 21;11(16):3126–35. doi: 10.1002/cam4.4677 (PMC9385586; doi:10.1002/cam4.4677)

## Slide 1
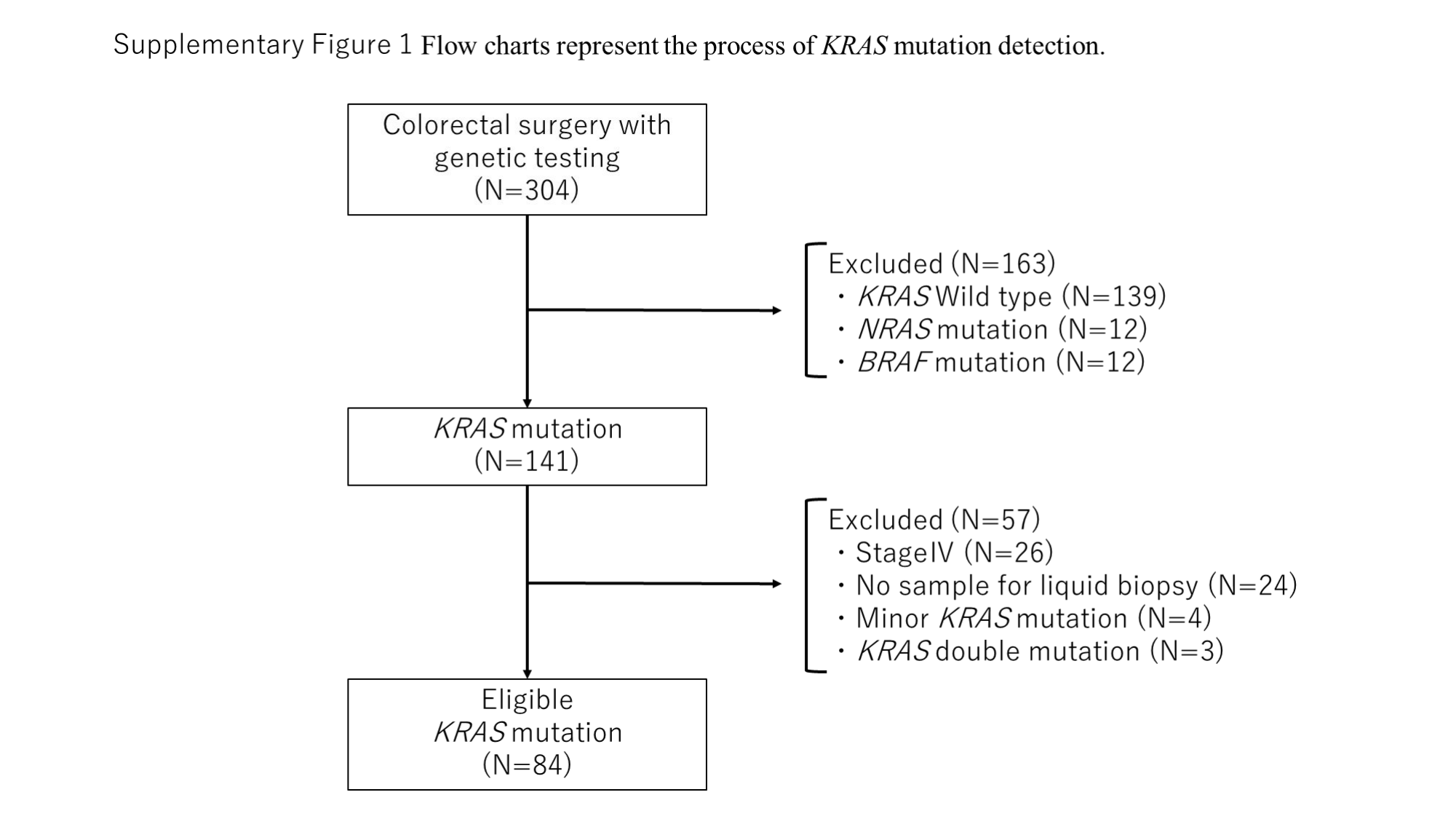

Supplement: Supplementary file 1 — Figure 1 [file CAM4-11-3126-s002.pptx]

## Slide 1
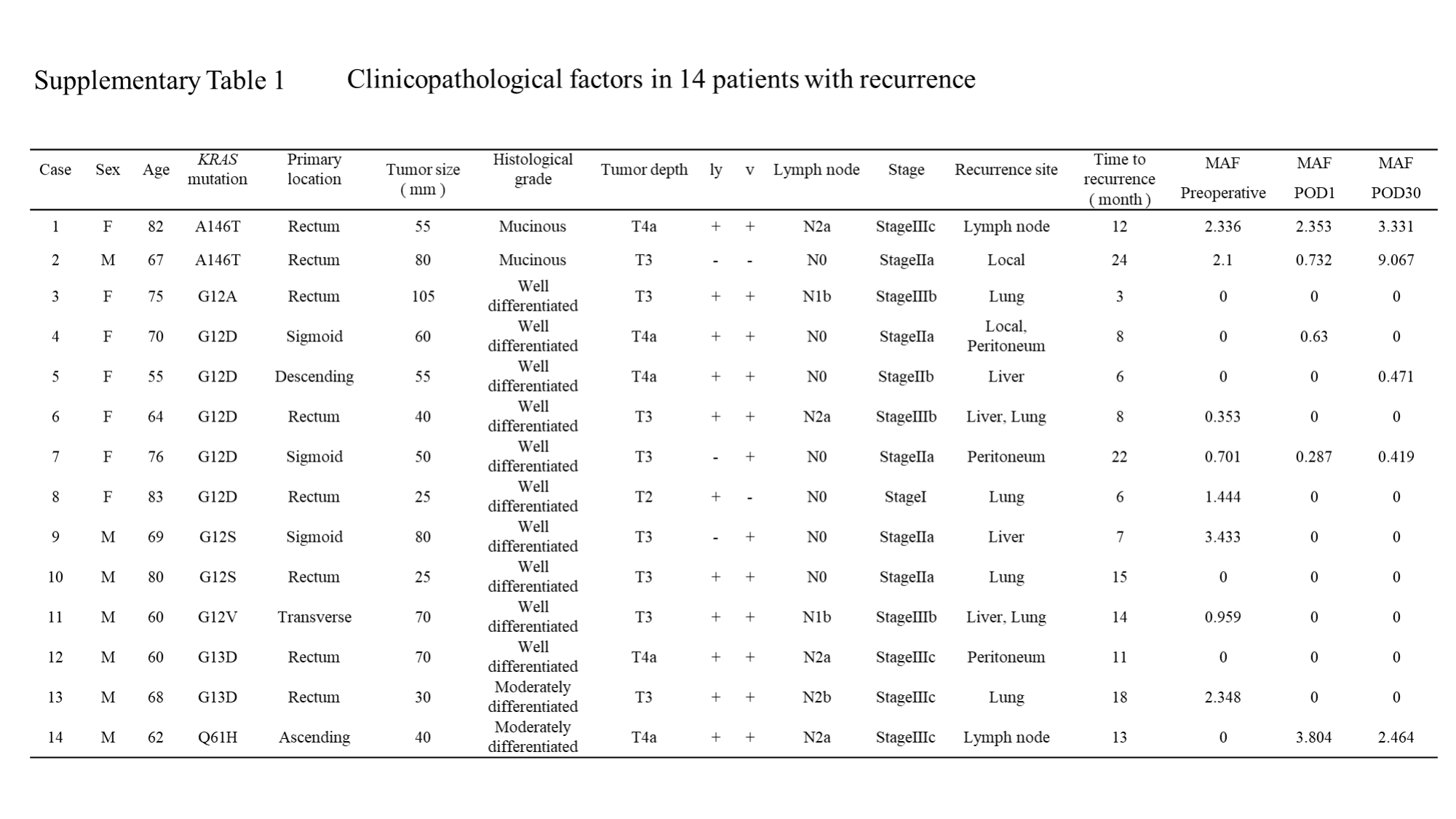

## Slide 2
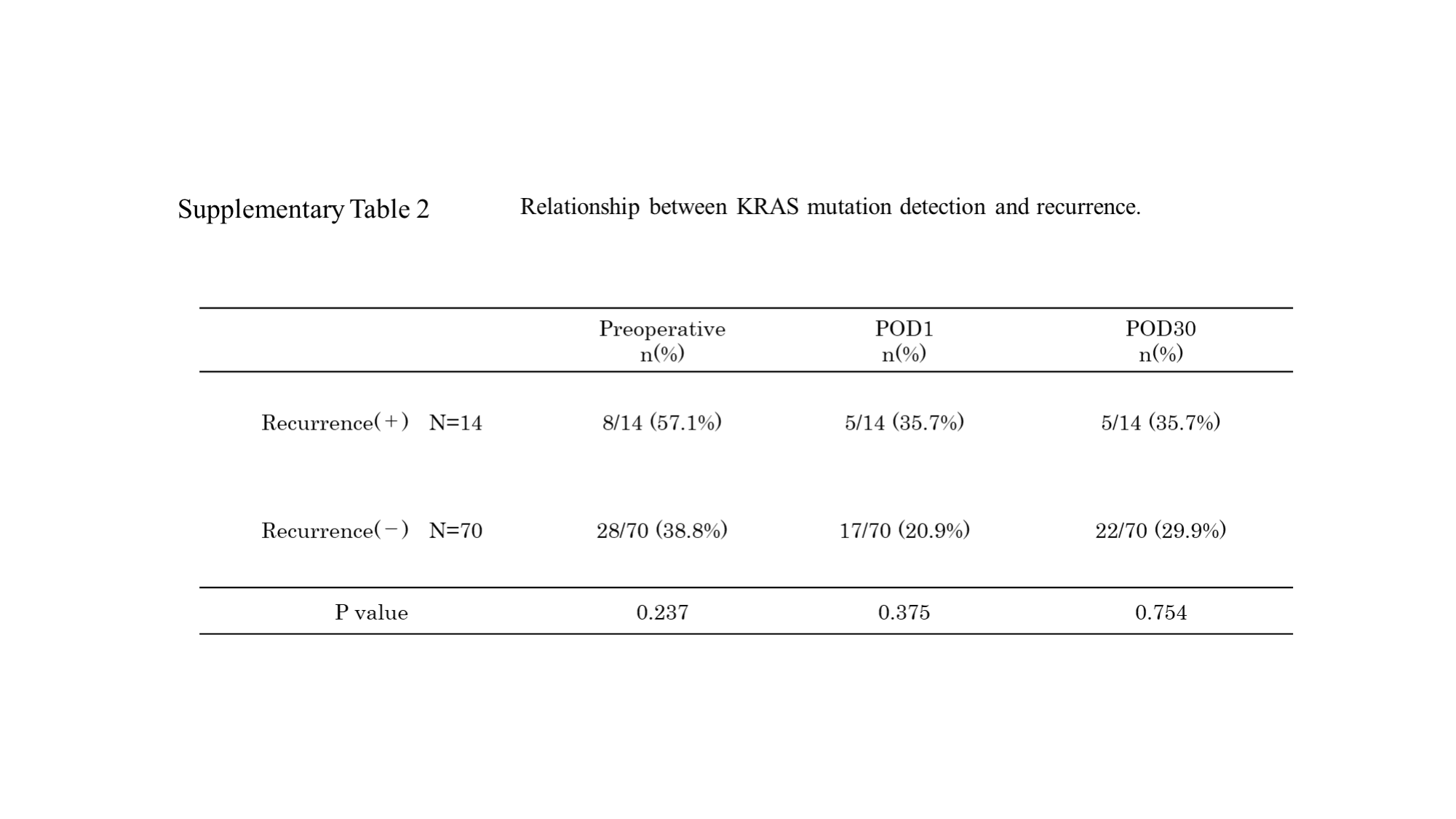

Supplement: Supplementary file 2 — Table 1 [file CAM4-11-3126-s001.pptx]
